# New perspectives for teaching Culture of Care and their strengths and challenges

**DOI:** 10.1177/00236772221127352

**Published:** 2022-10-06

**Authors:** Jordi L. Tremoleda, Angela Kerton, Hibba Mazhary, Beth Greenhough

**Affiliations:** 1 Blizard Institute, Barts and the London School of Medicine, UK; 2 Biological Services, Queen Mary University of London, UK; 3 The Learning Curve (Development) Ltd, UK; 4 School of Geography and the Environment, Oxford University Centre for the Environment, University of Oxford, UK

**Keywords:** Training, communication, engagement, storytelling, empathy

## Abstract

Nurturing a culture of care remains a key strategy and needs to be well integrated in the education programmes for laboratory animal professionals. Addressing attitudes is a complex task that must ensure reflective learning approaches. Teaching strategies must facilitate a safe space to talk openly about emotions and caring responsibilities. We reflect on two training initiatives focusing on culture of care. Firstly, the ‘Care-full Stories’ tool, which uses fictionalised prompts (storytelling) to encourage participants to share their own stories from working in animal research. Feedback on its impact on establishing a safe space for sharing experiences and the importance of appreciating diverse perspectives between staff is discussed. Secondly, we provide feedback on the development of training approaches on animal research integrity and culture of care with low- middle-income international communities. Strategic targets addressing the multicultural diversity of the communities, recognising their specific needs and their access to resources, must be well defined. It is important to acknowledge the interconnection between people, animals and their shared natural environment when defining the culture of care concept and addressing the teaching approaches. We discuss both the positive outcomes and challenges of these two learning experiences to support innovation when planning tools for teaching culture of care. Accounting for ‘how’ and ‘where’ the training will be delivered remains key to its successful uptake and local sustainability. Supporting improved educational tools to ascertain why caring has an impact on our professional lives will have a direct impact on the wellbeing of laboratory animal professionals worldwide.

## Points forts et défis des nouvelles perspectives d’enseignement de la culture de soins Résumé

Renforcer une bonne culture de soins reste une stratégie clé à bien intégrer dans les programmes de formation des professionnels exerçant dans les laboratoires utilisant des animaux. Faire face aux attitudes reste une tâche complexe et qui doit assurer des approches d’enseignement basées sur la réflexion. Les stratégies d’enseignement doivent encourager un espace sûr permettant de parler ouvertement d’émotions et de responsabilités en matière de soins. Nous examinerons deux initiatives de formation centrées sur la culture de soins. Il s’agit d’abord de l’outil « Care-full stories » qui repose sur des incitations fictives (narration) pour encourager les participants à partager leur propre expérience de travail dans la recherche animale. La rétroaction sur son impact en termes d’établissement d’un espace sûr pour partager des expériences, et prendre en compte les diverses perspectives du personnel, sera discutée. Nous aborderons ensuite le développement d’approches de formation sur l’intégrité et la culture de soins dans la recherche animale dans les communautés internationales à faibles et moyens revenus. Les cibles stratégiques prenant en compte la diversité multiculturelle des communautés, reconnaissant leurs besoins particuliers et leur accès aux ressources doivent être bien définies. Il est important de reconnaître l’interconnexion qui existe entre les gens, les animaux et leur environnement naturel partagé pour définir le concept de culture de soins et envisager les approches d’enseignement. Nous discuterons des résultats positifs et des défis issus de ces deux expériences de formation pour soutenir l’innovation dans le cadre de la planification des outils d’enseignement de la culture de soins. Rapporter « comment » et « où » la formation sera fournie reste un aspect clé pour assurer l’adoption et la durabilité locale. Soutenir de meilleurs outils de formation assurant pourquoi la culture de soins est importante dans nos vies professionnelles aura un impact direct sur le bien-être des professionnels qui travaillent dans les laboratoires utilisant des animaux.

## Neue Perspektiven für die Vermittlung einer Kultur der Fürsorge und ihrer Stärken und Herausforderungen Abstract

Die Förderung einer guten Kultur der Fürsorge (Culture of Care) gilt unverändert als eine Schlüsselstrategie, die gut in die Ausbildungsprogramme für Labortierpfleger integriert werden muss. Die Thematisierung von Einstellungen ist nach wie vor eine komplexe Aufgabe, bei der reflektierende Lernansätze gewährleistet werden müssen. Lehrstrategien müssen einen sicheren Raum schaffen, in dem offen über Emotionen und die Verantwortung für die Pflege gesprochen werden kann. Wir beschäftigen uns hier näher mit zwei Schulungsinitiativen, die sich auf die Kultur der Fürsorge konzentrieren. Erstens geht es um das Tool „Care-full Stories“, das die Teilnehmer anhand von fiktionalen Vorgaben (Storytelling) dazu anregt, ihre eigenen Geschichten aus der Tierforschung zu erzählen. Es wird Feedback zu seinen Auswirkungen auf die Schaffung eines sicheren Raums für den Erfahrungsaustausch und die Wertschätzung der unterschiedlichen Perspektiven der Mitarbeiter diskutiert. Zweitens werden wir Feedback zur Entwicklung von Schulungskonzepten zur Integrität in der Tierforschung und zur Kultur der Fürsorge in internationalen Gemeinschaften mit niedrigem und mittlerem Einkommen liefern. Strategische Ziele, die der multikulturellen Vielfalt der Gemeinschaften Rechnung tragen und ihre spezifischen Bedürfnisse und ihren Zugang zu Ressourcen berücksichtigen, müssen genau definiert werden. Bei der Definition des Konzepts der „Kultur der Fürsorge “und der Lehrprogramme ist es wichtig, die Verbindung zwischen Menschen, Tieren und ihrer gemeinsamen natürlichen Umgebung zu berücksichtigen. Wir werden die positiven Ergebnisse und Herausforderungen dieser beiden Lernerfahrungen diskutieren, um Innovationen bei der Planung von Lehrmitteln für die Kultur der Fürsorge zu unterstützen. Die Berücksichtigung des „Wie “und „Wo “der Ausbildung ist der Schlüssel für eine erfolgreiche Akzeptanz und lokale Nachhaltigkeit. Die Förderung besserer Lehrmittel, um zu erkennen, inwiefern Fürsorge unser Berufsleben beeinflusst, wird sich direkt auf das Wohlergehen von Labortierexperten weltweit auswirken.

## Nuevas perspectivas para la enseñanza de la cultura del cuidado y sus puntos fuertes y retos Resumen

Fomentar una buena cultura del cuidado continúa siendo una estrategia fundamental y debe estar bien integrada en los programas de formación de los profesionales que trabajan con animales de laboratorio. Abordar las actitudes sigue siendo una tarea compleja y debe garantizar enfoques de aprendizaje que hagan reflexionar. Las estrategias de enseñanza deben facilitar un espacio seguro donde poder hablar abiertamente sobre las emociones y las responsabilidades del cuidado. Reflexionaremos sobre dos iniciativas de formación centradas en la cultura del cuidado. En primer lugar, observemos la herramienta «Care-full Stories», que utiliza indicaciones ficticias (storytelling) para animar a los participantes a compartir sus propias historias de trabajo en la investigación con animales. Se debatirán los comentarios sobre su impacto a la hora de crear un espacio seguro para compartir experiencias y apreciar las diversas perspectivas entre el personal. En segundo lugar, proporcionaremos información sobre el desarrollo de enfoques de formación respecto a la integridad de la investigación con animales y la cultura del cuidado con las comunidades internacionales de ingresos medios bajos. Se deberán definir bien los objetivos estratégicos que aborden la diversidad multicultural de las comunidades, reconociendo sus necesidades específicas y su acceso a los recursos. Es importante reconocer la interconexión entre las personas, los animales y el entorno natural que comparten a la hora de definir el concepto de cultura del cuidado y al abordar diversos enfoques pedagógicos. Hablaremos sobre los resultados positivos y los retos de estas dos experiencias de aprendizaje para fomentar la innovación a la hora de planificar las herramientas de enseñanza para la cultura del cuidado. Considerar el «cómo» y el «dónde» se impartirá la formación sigue siendo clave para el éxito de la aceptación y la sostenibilidad local. Respaldar mejores herramientas educativas para saber por qué el cuidado repercute en nuestra vida profesional tendrá un impacto directo sobre el bienestar de los profesionales que trabajan con animales de laboratorio a nivel global.

## Culture of care in education: setting the scene, and challenges

Most contemporaneous regulatory and educational frameworks require the implementation of a harm–benefit assessment to ensure appropriate ethical justification for the use of animals in research^1,2^ and, importantly, to ensure the best care is provided and animal welfare is taken into account if non-animal alternatives are not available. Yet the transition from a focus on legal compliance to a more holistic approach that emphasises caring and empathetic attitudes in animal research work remains complex.^3^ The critical integrity of the research culture, excessive bureaucracy and hierarchical systems that prioritise compelling research and educational outcomes^4^ constantly challenge the development of more supportive working environments, risking disengagement and emotional dissonance amongst staff, which could directly impact on their capacity to care for other colleagues and the animals they are responsible for.^5,6^ The recent challenges created by the COVID-19 pandemic have also highlighted the need for better support to meet the mental and physical health needs of staff working with laboratory animals.^7,8^ Therefore, further interventions to promote a supportive, empathetic working environment and nurture a culture of care that supports both staff and animal wellbeing are clearly necessary.

Education continues to be instrumental in driving attitude changes and good practice in our work; thus, it is important to continue supporting innovative approaches to promoting and developing institutional cultures of care. Current regulatory guidelines acknowledge the role of education in establishing a solid foundation that should evolve into a long-term caring, responsible ethos and the professional commitment of researchers working with animals.^9–12^ Educational programmes are expected to seed culture of care practices in our researchers and staff; yet these programmes must also account for the varied professional roles and responsibilities these individuals hold, as well as respecting individual ethical and cultural values, different understandings of animal sentience and empathetic approaches towards other colleagues and the animals under their care. Planning a strategy for ‘teaching a culture’ remains complex, and needs to combine commitment to ensuring animal care and welfare, good quality science and an emphasis on professionalism, as well as aligning with the 3Rs (i.e. replacement, reduction and refinement).^13–15^

Current educational strategies mostly target professional competence and the development of technical skills and legal compliance; they offer limited scope for reflective learning and student engagement with peer argumentation and open debates, particularly between colleagues with different roles and responsibilities within a team.^16^ Active engagement and openness are instrumental to supporting a deeper understanding of how a range of different skills and individual responsibilities need to be valued and safeguarded in order to nurture a culture of care. It is important to incorporate the contemporaneous relevance of the 3Rs along with the multifaceted concepts of wellbeing (e.g. positive and negative affective states), experimental design strategies and, importantly, acknowledging the role played by different individuals,^17^ including researchers, animal technologists and managers, in supporting such empathetic and caring environments.^18^ It is also important to be sensitive and adaptable to the needs of diverse communities targeted, particularly when addressing international schemes.

Unfortunately, current curriculums and existing syllabuses are typically quite dense, and there is a general reluctance to implement further change. The intended learning objectives offer comprehensive delivery targets, but could benefit from further critical reflection on and assessment of the teaching methods used to deliver those outcomes.^19^ For example, while a person might understand how to meet the technical provisions laid out within the legislation, they may not always understand how their own practical behaviours and actions can contribute to, or impact on, standards of science or animal welfare and the ability to meet the wider requirements of the regulations.

That said, there have been remarkable efforts from the laboratory animal science and welfare community towards developing more participative and engaging training programmes.^20^ These offer a template for developing innovative approaches to promote deeper discussion of, for example, the relationship between emotions and caring responsibilities. They offer examples of how we might encourage staff to openly discuss how they feel about their work and any related challenges, and how such personal reflections can constructively build better trust and respect amongst all staff and in relation to the animals we work with. Contemporaneous training tools must be integrative, facilitating cross cultural and multidisciplinary discussion across all staff working with laboratory animals. This includes working to create physical and emotional spaces where staff can feel comfortable, where they are assured what they share will be held in confidence and not used in decisions about their contact or work, and that allow for all thoughts and voices to be heard. It is crucial for all levels of staff to be empowered and encouraged to communicate with each other, as this is key to embracing ‘a team sharing responsibility approach’, to maintaining and strengthening professional integrity and confidence in the work of staff.^21^ At the same time, while we can encourage and facilitate such discussions, we must also be mindful of not forcing people to share things they are uncomfortable making public, and respect people’s right not to share if they so choose.

## How to approach this

Our main objective in this article is to reflect on the necessity to strengthen the education of a culture of care. In particular, we will be reflecting on some ongoing experiences that are using novel training initiatives to support this.

In thinking about training to promote reflection on professional attitudes, it is important to embrace approaches that acknowledge how the already embedded ‘caring attitudes’ and excellent technical skills that most staff and researchers have will impact on the other less technical parts of their jobs, for example, communication between colleagues and management, dealing with emotions, balancing personal and professional responsibilities, and personal moral positions with respect to animal care and use. Importantly, training schemes need to embrace different professional responsibilities, avoiding any hierarchical settings, to support safe approaches for expressing failure and promoting positive perspectives.^22^

Specifically, creating space for reflection is crucial to capturing individual or collective experiences at work (e.g. presenting any monitoring or husbandry issues with the animals, sharing feelings on killing animals, expressing difficulties managing workloads, ethical challenges faced when carrying out duties), and to analysing and learning from those experiences. Team exploration and interactive reflection on how best to work together are also crucial, particularly for junior staff who need to feel supported and engaged with other staff, including senior management. Diversity and inclusivity are to be celebrated, along with individual contributions to teams, embracing the individual and collective impact on the overall institutional culture of care. Seeking global wellbeing for all the beings involved would guarantee a better culture of care, and better educational framework.

Bearing these points in mind, in what follows we discuss two training approaches we have used to create safe spaces for reflection as part of culture of care training initiatives. Firstly, we discuss the ‘Care-full Stories’ tool, which uses a storytelling approach as a reflective and participative training platform, initially working with UK-based animal research facilities. Secondly, we provide some reflective feedback on the outcomes and challenges when developing online training tools for low- middle-income countries (LMIC; according to the Organisation for Economic Co-operation and Development's Development Assistance Committee list, www.oecd.org/dac/financing-sustainable-development/development-finance-standards/daclist.htm), as part of supporting local capabilities in laboratory animal education in these countries. This latter approach is exemplified through two collaborative training projects with Peru and Thailand.

## Initiative 1: Storytelling as an approach to engaging in culture of care training

The Care-full Stories tool uses fictionalised prompts (storytelling) to encourage participants to share their own stories from working in animal research.^23^ Through this process it aims to build and acknowledge the emotional connections within the professional duties of professionals/researchers, sharing personal experiences to promote discussions on attitudes and challenges that may arise when working in the field of animal research. The tool has been designed and piloted in the UK, driven by a group of social science and humanities scholars linked to the Wellcome-funded Animal Research Nexus project (www.animalresearchnexus.org). Drawing on their work, and in particular how the participants they spoke to often told stories as a way of sharing particularly emotionally or ethically challenging aspects of their work, the scholars saw how storytelling could offer an alternative to teaching a culture of care. Storytelling is a practice that spans diverse cultures and nationalities. If you think back to your own upbringing, one of the most impactful ways of learning is believed to be through storytelling. Most of us can remember our favourite bedtime story or fairytale. Therefore, we might hypothesise that storytelling offers a useful platform through which to facilitate educational and learning experiences.

Storytelling also addresses more practical challenges. In an already jam-packed curriculum (and work diary) it can be challenging for people to find the time to talk transparently, honestly and in detail about questions such as what constitutes good care and how this can be practically applied within the laboratory animal science field. The struggle to ‘find time to talk’ may be further compounded by researchers and staff opting to not discuss their profession and details of their work outside the workplace for fear of judgement or repercussions from members of the general public, or even close family members who are not supportive of the use of *in vivo* animal models for research purposes. By changing how we teach (e.g. by using story sharing methodologies) as opposed to what we teach (the culture of care is already a key learning objective incorporated into many training programmes), we can make a significant intervention into cultures of care in laboratory animal work.^24^

**Figure 1.**
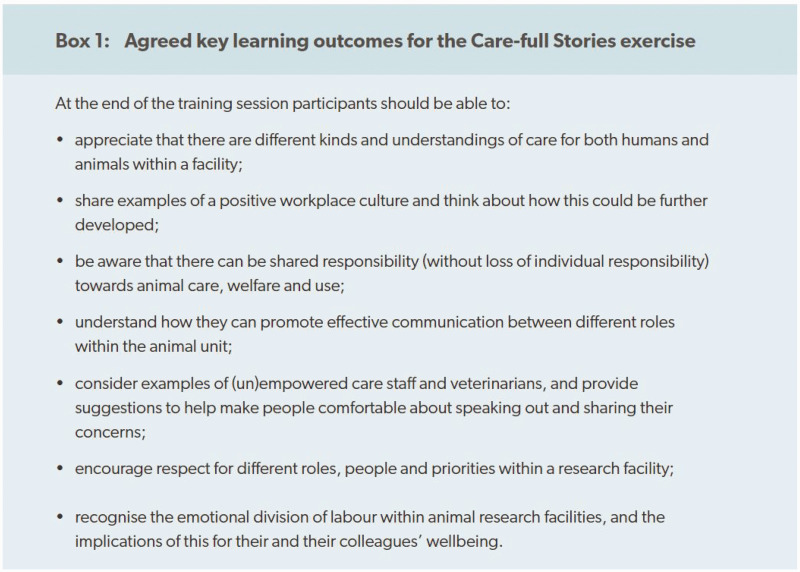
Agreed learning outcomes: Care-full Stories.

In order to ensure any new training resource would be aligned with existing training programmes and needs, we took a collaborative approach to research development, working with an advisory group of stakeholders who represented the diversity of the UK animal research community, including managers, researchers, trainers and animal care staff, as well as representatives of non-governmental organisations and private- and public sector organisations. During an early preparatory phase of the project, the stakeholders agreed on a list of learning outcomes that were to be achieved by participants who attended the workshop (Figure 1). These learning outcomes, in combination with stories and experiences collected during interviews(*n* = 156) conducted by members of the Animal Research Nexus project and focus group discussions at animal research facilities, were used to create a brief for a professional script writer, who worked with us to develop three short scripts, each around two to three pages, which could be read out (performed) in a workshop as a starting point for facilitating discussion.^23^

Each workshop began with the facilitator providing an overview of the aims and expected learning outcomes for the session (Figure 1). During this opportunity to set the scene, it was vital to establish a safe space by requesting that participants keep the specific details and examples shared within the workshop confidential. This enabled trust to be quickly established by all those committed to the training. We also found it beneficial to include a short ice-breaking activity (several examples are available in the training resource pack) to establish a relaxed and friendly tone for the workshop. This was then followed by a script reading and group discussion. In the build-up to the workshop (generally a few days before), facilitators were encouraged to ask for volunteer readers for the roles in each script and these volunteer readers were supplied with copies of the scripts in advance of the workshop date. We found that this activity worked best when volunteers read a role that differed from their own. To facilitate open reflection and sharing of opinions, there are recommended questions for discussion at the end, but these can be easily adapted based on the group dynamics to allow the conversation to flow and explore topics beyond those suggested.

**Figure 2.**
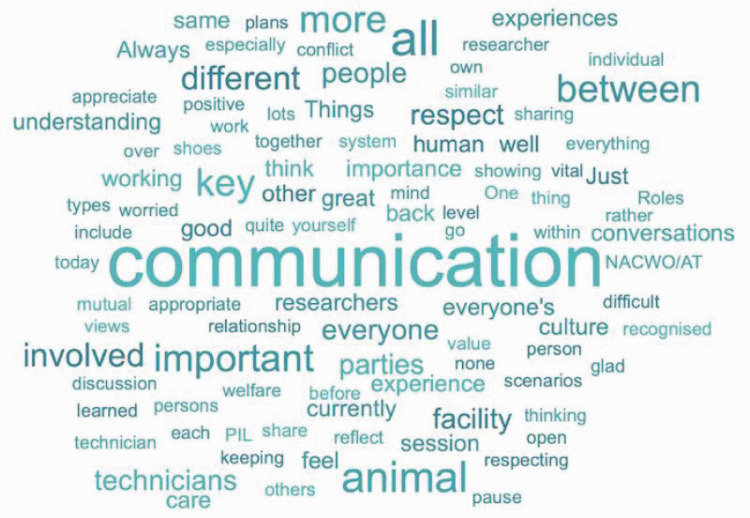
Word cloud from the prompt ‘One thing I have learned from today’s session is …’.

**Figure 3.**
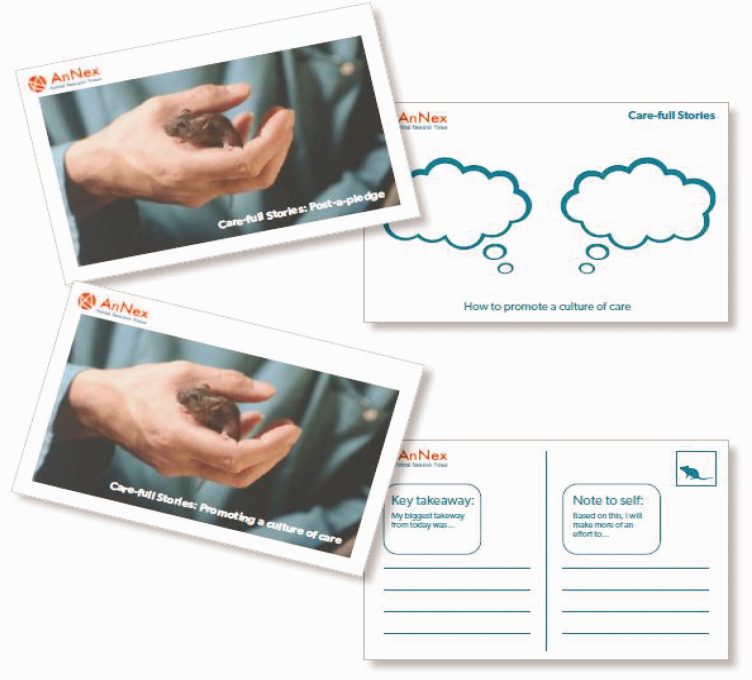
Post a pledge postcard design for the Care-full Stories training resource.

In our pilot sessions to date (*n* = 10), as well as in demonstrations of the resource at industry conferences and events, we have found that Care-full Stories has opened up new spaces for thinking about the research culture and offered a good starting point from which to explore dealing with change, which can be unsettling for many researchers when the status quo is challenged. At the end of the session participants were encouraged to reflect on what they had learned, posting comments to an online whiteboard or sharing thoughts to be added to a flipchart or similar in person. To give an indication of the kind of themes that emerged from the discussions, Figure 2 offers a summary of responses to the prompt ‘One thing I have learned from today’s session is …’. To reinforce future commitment and knowledge acquisition, the training resource contains useful examples of ‘pledge postcards’, which participants complete at the end of the session and facilitators arrange to post back to them a few months later. This was intended to be a useful prompt or aide memoire to put learning into action once participants have returned to the workplace (Figure 3).

Feedback indicated that the workshops were very successful in highlighting the importance of open communication and the need to have empathy and respect for the wide range of roles within the animal research sector. Participants particularly enjoyed the interactive nature of the role playing and embraced the chance to share stories. Comments included reflections on the ‘eye opening’ insights into the culture of care and appreciation of the valuable opportunity for detailed and open discussion. This helped with exploring other participants’ roles, addressing questions such as who has responsibility for generating a good culture of communication, and the importance of respecting different viewpoints in an animal research facility. Despite the seriousness of the topic being taught, the storytelling approach was found to be ‘fun and engaging’. Constructive feedback also revealed that, although the exercise did work successfully online, some of the theatrical and role-playing elements were lost in the virtual setting (such as switching seats and using props). We are currently developing three more scripts to explore more diverse setting and scenarios, as well as planning further pilots.

We encourage all those involved in providing training on a culture of care to pilot small group workshops using the resources available from the Care-full Stories project. The teaching experience and results will be not only be enjoyable and valuable, but could work to create safe and reflective spaces that might encourage the sharing of stories, experiences and concerns amongst those with different roles and responsibilities within an animal research facility, thereby supporting the development of, and investment in, a shared culture of care.

## Initiative 2: Training approaches to promote a culture of care in international communities

Our first example was developed in the UK, and while we are working to test and further develop these resources for use with a wider global audience, we are also aware that creating training materials on animal and staff care and welfare, which can be used across divergent sites and spaces of animal research, brings with it additional challenges. How do we work towards an arguably shared desire for good animal welfare across the global animal research sector, while recognising diverse local values, practices and capacities?

In this second example we reflect on the experiences gathered from the development of training on animal research integrity and culture of care for two LMICs: an online training resource for planning preclinical animal studies, as part of a larger set of animations for trauma research developed in collaboration with Thai medical institutions; and an online module on research ethics and scientific integrity, with a network of academic institutions in Peru. These projects, which were supported by the Global Challenges Research Fund Scheme–UK Research and Innovation (GCRF-UKRI^25^) were set up to build capacity and expertise amongst local researchers with respect to the care and welfare of laboratory animals, research integrity, and the 3Rs, which in turn support a broader agenda of raising awareness globally of the need to establish a good culture of care.

A major consideration when engaging with schemes such as GCRF is to clearly identify the Sustainable Developmental Goals (SDGs) that will be targeted in your programme, thereby directly aligning your work with the SDG commitment to interventions that are sustainable and build capacity within local communities (see UNESCO SDGs^26^). Our proposed culture of care learning platform has established goals that are aligned to improve the quality of education and training – particularly within higher educational programmes, which have a direct impact on local innovation and development – and through these means, support better harmonisation with international standards of animal care and welfare. For our programme with Peruvian institutions, it was instrumental to acknowledge, and thus protect, the unique local biodiversity of their country, particularly in the Andean communities. It is critically important to strike the right balance between international harmonisation, through promoting a shared commitment to animal care and welfare, and respect for local cultures, value systems and ethical principles, such as ancestral obligations to protect and preserve biodiversity.

Bearing these considerations in mind, our project set out four key objectives: a) to promote international standards for animal husbandry and welfare through knowledge transfer lectures and Q&A discussions; b) to support local leadership for policy development on the 3R principles for use of animals in research – including research outside the lab, for example, in conservation; c) to establish collaborative training/research projects between different institutions as an opportunity to ensure sustainability and well-balanced partnerships between UK/EU and LMIC local institutions; and d) to promote the implementation of a local network to establish further training/educational multidisciplinary programmes, particularly within the schemes of biomedical/conservation and population studies. This latter scheme is particularly important to ensure mid- to long-term programmes for collaborative research and training following our introductory networking workshop.

As part of these examples, we supported the development of an online module, Research Ethics and Scientific Integrity, in Peru, which covered animal research and welfare, harm and benefit assessments, research reproducibility, and contemporaneous regulatory perspectives on the 3Rs and culture of care. These online training platforms were attended by over 80 researchers, academic staff, technical staff and managers, representing a diverse learner community from higher education institutions across the country. The main aim of the module was to strengthen theoretical and applicable knowledge for research and innovation, and to build a better awareness of and engagement with developing a good culture of care.

**Figure 4.**
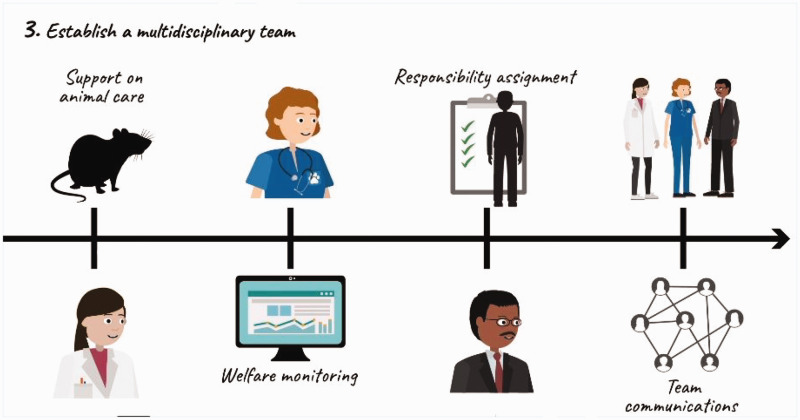
Example of the visuals and information developed as part of the preclinical animation, with a key focus on international standards for undertaking animal research, covering the 3R principles for a responsible approach to animal research to ensure animal welfare, standards in experimental design and transparency in reporting.

As part of another training collaboration with medical institutions in Thailand, we developed a set of online training platforms to build further global expertise in trauma research. The project, also supported through a GCRF grant at Queen Mary University of London, aimed to strengthen research capacity in Thailand. Due to the challenges of the COVID-19 pandemic restricting opportunities for face to face teaching, the online training platform comprised four animated videos that offered guidance on a) planning preclinical animal studies; b) gaining informed consent; c) establishing a biobank; and d) creating and maintaining a database for a research study, all tailored towards the specific needs of those working in trauma research. The platform targeting the planning of preclinical animal studies provides a clear set of guidelines that should be considered when embarking on preclinical translational studies. These outline the implementation of the 3Rs and provide a set of steps including 1) the implementation of a clear hypothesis and experimental plan; 2) taking account of all legal and ethical considerations; 3) establishing a multidisciplinary team to support the care and welfare of animals and staff; 4) implementing good experimental practices; and 5) good reporting and transparent communication. These approaches are fundamental to both research integrity and a culture of care, specifically by ensuring the sharing of resources, individual and team expertise, and responsibilities and through encouraging collaboration and communication within and between institutions. Due to the translational nature of this particular training platform, it was crucial for the animal research work to be well integrated with the other clinically related platforms on patient consent and sample/data management. This case therefore offers useful insights into how a concern for promoting a culture of care within animal research work can also impact on and be impacted by broader institutional and disciplinary research environments. These platforms can be viewed freely on the institutional website: www.c4ts.qmul.ac.uk/main/latest-news/post/85-four-new-animated-videos-to-facilitate-quick-up-take-in-trauma-research-methodology (see Figure 4).

Acquiring feedback from such international training schemes can pose a range of challenges, as direct communication with attendees via online platforms can be more challenging that in-person training schemes. Furthermore, dissemination for further engagement is ongoing and, thus, we envisage acquiring further feedback in the near future. Interestingly though, as the online platforms were promoted via social/professional media, we received excellent support from health professionals worldwide, who retweeted the links for accessing these training resources. This social/professional media approach has proven to be a very successful route for international reach. To date, feedback gained has been very positive, particularly in commending the straightforward access to and uptake of the online video training material (e.g. ‘entertaining and useful for anyone in clinical research’).

When setting up such international training schemes it is important to consider key strategic points, accounting for impact, local capabilities and sustainability. With regard to impact, it is important to identify appropriate pathways to ensure that the developing country benefits from your proposed training, ensuring that the outcomes expected are realistic and appropriate to the particular needs and realities of your collaborating country. It is important that the benefits expected are directed to the communities and primarily relevant to them: identify who will be directly affected by the training; outline the direct benefits, even if small, as it is best to be realistic; and consider how many people will be affected by the progress. With this in mind, it is important to engage with the communities/collaborators at the early planning stages of the project. Systems are very different across countries, communities and institutions – bureaucracy can feel overwhelming and system logistics very challenging (e.g. fund transactions, appropriate access to informatics systems) so it is fundamental to rely on the expertise and feedback of collaborators.

Regarding sustainability, it is crucial to strengthen the inclusion of other institutional activities in the region and establish further agreements with local institutions, providing a clear outline on how these agreements will enhance local innovation and research capability. It is important to consider potential impacts at the individual, institutional and country levels – impacts at each level can be very valuable. The influence of key few individuals can sometimes be a more effective way of promoting change than trying to overhaul complex and embedded bureaucratic systems. Importantly, when thinking about long-term impacts, engaging with younger generations may be key, particularly in regard to the broader attitudes of empathy, diversity, equality and respect, which are fundamental pillars for establishing a good culture of care. From our experiences, the younger attendees showed great engagement and interest during the training, and interestingly, were mostly driven by their empathetic attitude toward any animals or plants used for research studies. They showed a remarkable interest in understanding how good care and welfare could be promoted as core components of culture of care.

Finally, the economic and social challenges for these LMICs remain a key concern for local researchers; economic and logistic support needs to be driven through local capabilities and truly collaborative programmes, not through a ‘direct unaccountable and unmanaged charity donation’. In our sessions, the lack of resources was highlighted by participants in all Q&A and discussion sessions. From our perspective, it is important to take a pragmatic approach; the knowledge and expertise that your training will bring are key assets, but we must ensure that our expectations are realistically harmonised with the local resources available and the actual needs of the local community. Innovation and creativity are not always directly related to economic support. Remember: small but well-grounded steps are likely to have a robust mid- to long-term impact.

From these experiences, we surmise that it is important to take a broader perspective on the teaching of culture of care that considers not only the animals and staff involved but also the natural and cultural (and political) environment they share. This highlights the importance of recognising the interconnections between people, animals, plants and their shared environment within both the culture of care concept and teaching approaches. Supporting better educational frameworks will guarantee better tools to ascertain why and how caring has an impact on our professional lives, promoting global wellbeing for all the beings involved.

## Conclusions

We opened this article by highlighting the need to transition culture of care training from a model focused on legal compliance towards a more holistic approach that emphasises caring and empathetic attitudes in animal research. We also noted the challenges posed by seeking to teach and facilitate such a transition in a pressurised, outcome-focused environment that provides little space for frank, open discussion and reflection, particularly between those with very different roles and responsibilities with respect to animal research (e.g. managers, researchers, trainers, veterinary staff and animal care staff, and potentially even patients and wider publics^27^). We have outlined two innovative approaches to teaching a culture of care as examples of how we might, through education, respond to these needs and challenges.

The first of these, developed in the UK, uses short story scripts to encourage debate, discussion and the sharing of experiences around an institutional culture of care amongst the different staff who work within an animal research facility, noting that this approach is particularly effective in highlighting the need for good communication and the importance of being able to ‘put yourself in someone else’s shoes’ to appreciate their perspective. In practice, working to create a safe space for these discussions to take place and the skills of the facilitator also play a key role.

The second example reflects on two experiences of teaching a culture of care in institutional settings outside Europe, looking at examples of online training platforms developed in collaboration with local research and higher education institutions in Peru and Thailand. These cases brought home the need to balance a shared commitment to promoting high standards of animal and staff care and welfare, with a sensitivity to local needs, resources and cultures, and an appreciation of how these in turn can shape what a culture of care is and how it may be best promoted and supported. These examples also stressed the need to focus on building local capacity and developing realistic and sustainable training programmes.

Collectively then, when developing training resources on the topic of culture of care, our examples stress the need to balance the focus on learning outcomes with attention to (i) *how* such materials are delivered, in particular the need to create space for reflective discussion where people can share experiences, and (ii) *where* such training is to be delivered, being sensitive to local needs, capacities and resources and to the local sustainability of any new training initiatives.

## Data Availability

This is a reflective essay discussing training platforms. No raw data are available.
